# Palliative enteral feeding for patients with malignant esophageal obstruction: a retrospective study

**DOI:** 10.1186/s12904-015-0056-5

**Published:** 2015-11-05

**Authors:** CW. Yang, HH. Lin, TY. Hsieh, WK. Chang

**Affiliations:** Division of Gastroenterology, Department of Internal Medicine, Tri-Service General Hospital, National Defense Medical Center, No 325, Section 2, Cheng-Kung Road, Neihu 11490, Taipei, Taiwan

**Keywords:** Esophageal cancer, Esophageal stent, Palliation, Nasogastric tube

## Abstract

**Background:**

Malignant esophageal obstruction leads to dysphagia, deterioration in quality of life, and malnutrition. Traditional bedside nasogastric (NG) tube placement is very difficult under these circumstances. However, endoscopically assisted NG tube placement under fluoroscopic guidance could be an alternative option for establishing palliative enteral nutrition. This study aimed to compare the clinical outcomes of enteral tube feeding and esophageal stenting for patients with malignant esophageal obstruction and a short life expectancy.

**Methods:**

Thirty-one patients were divided into 3 groups according to their treatment modality: NG tube (*n* = 12), esophageal stent group (*n* = 10), and supportive care with nil per os (NPO) (*n* = 9). Enteral nutrition, clinical outcomes, length of hospital stay, and median survival were evaluated.

**Results:**

There were no significant baseline differences among the groups, except in age. The tube and stent groups had significantly higher enteral calorie intake (*p* = 0.01), higher serum albumin *(p* < 0.01), shorter hospital stay (*p* = 0.01), and longer median survival (*p* < 0.01) than the NPO group. The incidence of dislodgement in the tube group was significantly higher than in the stent group (58 % vs. 20 %, respectively; *p* = 0.01). However, stenting costs more than NG tube placement.

**Conclusions:**

Palliative enteral feeding by NG tube is safe, inexpensive, and has a low complication rate. Endoscopically assisted NG tube placement under fluoroscopic guidance could be a feasible palliative option for malignant esophageal obstruction for patients who have a short life expectancy.

**Electronic supplementary material:**

The online version of this article (doi:10.1186/s12904-015-0056-5) contains supplementary material, which is available to authorized users.

## Background

Esophageal cancer has a high mortality rate because many patients are not diagnosed until the late stages of disease [1]. Malignant esophageal obstruction, the condition wherein a tumor obstructs the esophagus [2], always leads to dysphagia, which is one of the most troubling and unbearable symptoms of this condition. Diagnosed when the stenotic portion of the esophagus is less than 14 mm in diameter, dysphagia causes malnutrition and a deterioration in quality of life [3]. Initially, patients developing a malignant esophageal obstruction will have difficulty swallowing solid food. This can progress to difficulty swallowing semisolid food and, finally, difficulty swallowing saliva and water [4]. These patients often have a poor appetite, may experience significant weight loss, and are likely to develop nutritional compromise. A poor nutritional status is a significant prognostic factor for mortality in patients with esophageal cancer [5].

Not only are patients with malignant esophageal obstruction harmed as a result of nutritional compromise, the resulting cachexia accounts for profound adverse effect on patients’ quality and length of life [6]. Therefore, nutritional supplementation is important to improve the overall condition and quality of life of these and all patients with esophageal cancer [7]. For patients with terminal-stage esophageal cancer, the aim of nutritional therapy is palliative care, because the health-related quality of life of these patients is very poor. Typically, enteral feeding is considered superior to total parenteral nutrition because parenteral nutrition has a higher risk of complications and a higher expense [8]. However, for patients with advanced-stage esophageal cancer, establishment of enteral nutrition is difficult.

The traditional bedside blind-passage method of nasogastric (NG) tube insertion fails in most cases of malignant esophageal obstruction. In addition, percutaneous endoscopic gastrostomy (PEG) might not be possible if the endoscope cannot pass through the stenosis. An alternative, esophageal stent placement improves dysphagia quickly [9] and provides better long-term relief [10]. Hence, esophageal stenting is considered an option for esophageal cancer patients with severe dysphagia combined with a short life expectancy or recurrent tumor growth after cancer treatment [11]. For these patients, endoscopy-assisted NG tube placement might be a suitable option for establishment of enteral nutrition. In this study, we aimed to retrospectively evaluate and compare the clinical outcomes of three palliative treatments: nutritional palliation with NG tube placement under fluoroscopic guidance, esophageal stenting, and a nil per os (NPO) regimen.

## Methods

### Patient selection

We reviewed the cases of 62 patients with malignant esophageal obstruction from 2003 to 2013. Thirty-one patients who underwent percutaneous endoscopic gastrostomy or surgical gastrostomy or jejunostomy were excluded, leaving 31, all of whom had been diagnosed with advanced-stage esophageal cancer requiring palliative treatment owing to metastasis or poor medical condition (unfitness to undergo surgery or chemotherapy). All patients had dysphagia, causing nutritional compromise and deterioration in quality of life, the severity of which was assessed by a dysphagia score. All patients’ Eastern Cooperative Oncology Group (ECOG) performance score was 4.

Endoscopically assisted esophageal stenting was the first treatment considered for all patients. Esophageal metallic stenting was not reimbursed by our health-insurance system in Taiwan. If patients refused a stent due to economic problems or their own choice, endoscopically assisted NG tube placement, which was covered by our health-insurance system, was suggested. If the patient could not undergo either stenting or NG tube placement, supportive palliative medical care was recommended, and the patient was placed on an NPO regimen. Supportive care with fluid hydration and minimal amount of oral liquid diet and water were performed in the NPO group.

### Data collection

For each patient, data were collected on age, sex, body mass index, nutritional condition (serum albumin at baseline and 3–4 weeks later), tumor characteristics (location, stage, and histology) (Table 1), presence of fistula, daily enteral caloric intake (on days 1, 3, 7, and 14 after the start of treatment), daily intravenous fluid volume (on the day prior to the procedure and days 1, 3, 7, and 14 afterward), complications, duration of hospital stay, and median overall survival (Table 2). Esophageal tumor stage was determined according to the 7th American Joint Committee on Cancer (AJCC) staging system.

**Table 1:** Patients’ characteristics

	NG tube	Esophageal stent	NPO	*P*-value
	(*n* = 12)	(*n* = 10)	(*n* = 9)	
Age in years	72.6 ± 14.1	58.0 ± 9.4	61.4 ± 7.8	0.01
Gender (M/F)	10/2	9/1	8/1	0.88
BMI	19.0 ± 3.3	18.1 ± 2.6	17.4 ± 2.6	0.45
Cancer stage				0.18
III	2	0	0	
IV	10	10	9	
Histology				0.07
SCC	9	10	9	
ADC	3	0	0	
Dysphagia score	3.6 ± 0.5	3.1 ± 0.6	3.8 ± 0.4	0.02
3	5	8	2	
4	7	2	7	
Fistula				
Yes	1	6	0	<0.01
No	11	4	9	
Albumin (g/dL)	2.8 ± 0.4	2.8 ± 0.5	2.5 ± 0.2	0.15
Indication for palliation				0.05
Metastasis	10 (83 %)	10 (100 %)	5 (56 %)	
Poor medical condition	2 (17 %)	0 (0 %)	4 (44 %)	

*NG tube* nasogastric tube, *NPO* nil per os, *SCC* squamous cell carcinoma, *ADC* adenocarcinoma

**Table 2:** Clinical outcomes and complications

	NG tube	Esophageal stent	NPO	*P*-value
	(*n* = 12)	(*n* = 10)	(*n* = 9)	
Albumin, g/dL	3.1 ± 0.5	3.0 ± 0.5	2.1 ± 0.4	<0.01
EN intake, dL/day	11.8 ± 4.9	10.9 ± 3.5	3.6 ± 1.9	0.01
IV fluid volume, dL/day	9.9 ± 4.9	9.4 ± 3.7	13.5 ± 3.3	0.08
LOH, days	19 ± 15	12 ± 11	39 ± 18	0.01
Survival, days	122^a^	133^a^	51	<0.01
Complications	22	21	9	
Aspiration pneumonia	7^b^	5^b^	9	0.38
Dislodgment	7^c^	2^c^	0	0.01
Chest pain	2	3	0	0.21
Perforation	0	0	0	
Hemorrhage	0	0	0	

^a^ *p* = 0.56 between NG tube group and esophageal stent group. ^b^ *p* = 0.63 between NG tube group and esophageal stent group. ^c^ *p* = 0.01 between NG tube group and esophageal stent group. *NG tube* nasogastric tube, *NPO* nil per os, *EN* enteral nutrition, *PN* parenteral nutrition, *LOH* length of hospital stay, *IV* intravenous

### Endoscopy-assisted NG tube placement

Patients were placed in the left lateral position for NG tube placement. Oxygen saturation, blood pressure, and heart rate were monitored during the procedure. All patients received a 12-Fr polyurethane feeding tube 114 cm in length (Entube; Rusch, Duluth, GA, USA). The tube was placed according to one of two methods: the guidewire method and the push method.

**Fig. 1:**
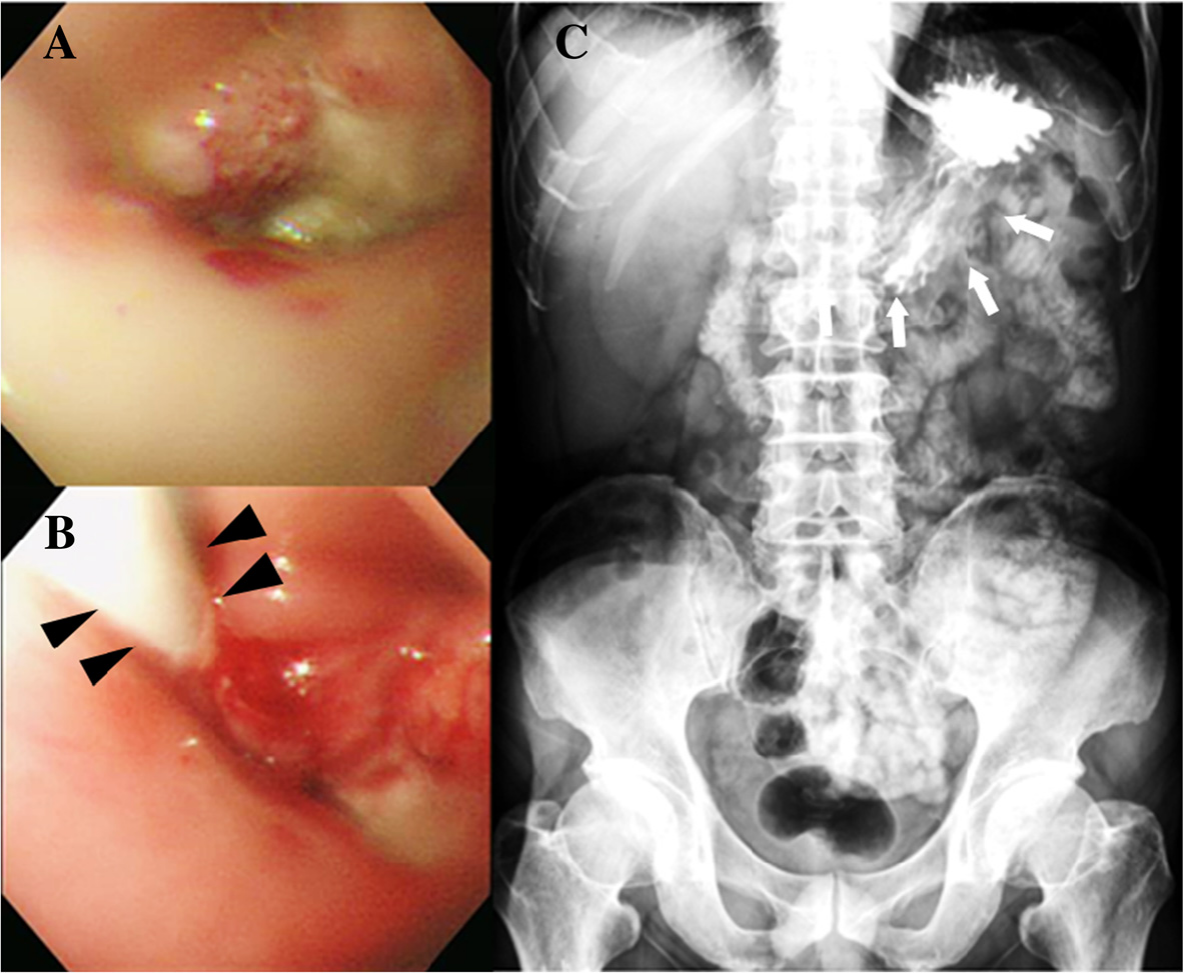
Representative images before and after endoscopy-assisted nasogastric tube placement (**a**) Esophagogastroduodenoscopy demonstrating nearly total obstruction of the esophagus by a tumor. **b** NG tube (black arrowhead) passed through the site of the obstruction. **c** Abdominal plain-film radiograph showing that the feeding tube is in the stomach (white arrow); reconfirmed using contrast medium

In the guidewire method, the weighted tip of the feeding tube was cut, and the outer wall of the tube was lubricated with topical lidocaine (Xylocaine Jelly; AstraZeneca, London, UK). The NG tube was flushed with water, loaded with a guidewire (Hydra Jagwire; Boston Scientific, Natick, MA, USA), inserted through the nostril, and passed into the esophagus. A gastroscope was introduced into the site of the stenosis (Fig. 1a).

In the push method [12], the NG tube, loaded with a guidewire, was directly introduced through the stenosis caused by the malignant esophageal obstruction. Initially, the guidewire was introduced through the stenosis under fluoroscopic guidance. Then, the NG tube was advanced through the stenosis site directly along the guidewire (Fig. 1b).

To confirm the position of the tube, contrast medium was injected through the NG tube under fluoroscopy. The gastroscope was withdrawn slowly out of the mouth with the feeding tube kept in place to prevent retrograde migration. The guidewire was removed after completion of the procedure. An abdominal plain-film radiograph was obtained to reconfirm the correct position of the feeding tube in both methods (Fig. 1c).

### Esophageal stenting

The malignant esophageal obstruction was assessed using upper gastrointestinal endoscopy before esophageal stent placement. First, a topical aerosol spray of lidocaine hydrochloride anesthetic was administered to the patient’s pharynx. A guidewire was introduced through the endoscope (GIF-Q260; Olympus, Tokyo, Japan), past the obstruction site, and into the distal part of the esophagus or stomach. A covered metallic stent (Ultraflex; Boston Scientific) was engaged by a guidewire under fluoroscopic guidance. The esophageal stent length of 10, 12, or 15 cm was selected based on the tumor size and length. After placement, the stent was dilated and deployed under fluoroscopic monitoring.

### Statistical analysis

SPSS software version 20.0 (IBM, Armonk, NY, USA) was used for all statistical analyses. One-way ANOVAs were used to analyze the relationships between the three groups. Student’s *t*-test, the *χ*^2^-test, and Fisher’s exact test were used to analyze the relationship between the NG group and the stent group. Overall median survival was calculated from the date of malignant esophageal obstruction diagnosis until death or the last follow-up. Certain parameters, including age, presence of fistula, and dysphagia score, were entered into a Cox regression model to analyze their relative prognostic importance and to plot survival curves, which were tested by the log-rank test. *p* < 0.05 was considered statistically significant for all analyses. The clinical data were obtained from our database which had been established in a double blind process. In addition, the Institutional Review Board of the Tri-Service General Hospital approved the use and analysis of clinical data (IRB: 2-104-05-030) (Additional file 1).

## Results

### Baseline characteristics and duration of hospital stay

The 31 patients ranged in age from 38 to 88 years with a mean of 64.7 years. There were 27 men and 4 women. When malignant esophageal obstruction was diagnosed, the cancer was AJCC stage IIIC in 3 patients and stage IV in the remaining 28. Patients were divided into three groups according to treatment modality: the NG feeding tube group (*n* = 12), the esophageal stent group (*n* = 10), and the group assigned to supportive care under an NPO regimen (*n* = 9) (Table 1). In the NG feeding tube group, the tube was inserted using the push method in 8 patients and the guidewire method in the remaining 23. There were no significant differences in baseline clinical features among the three groups, except in age (Table 1); the three groups were similar with regard to sex distribution, body mass index, dysphagia score, location of tumor, AJCC stage, tumor histology, and baseline serum albumin. The average duration of hospital stay was 19 days in the NG feeding tube group, 12 days in the esophageal stent group, and 39 days in the NPO group (*p* = 0.01; Table 2).

### Daily enteral caloric intake and serum albumin

**Fig. 2:**
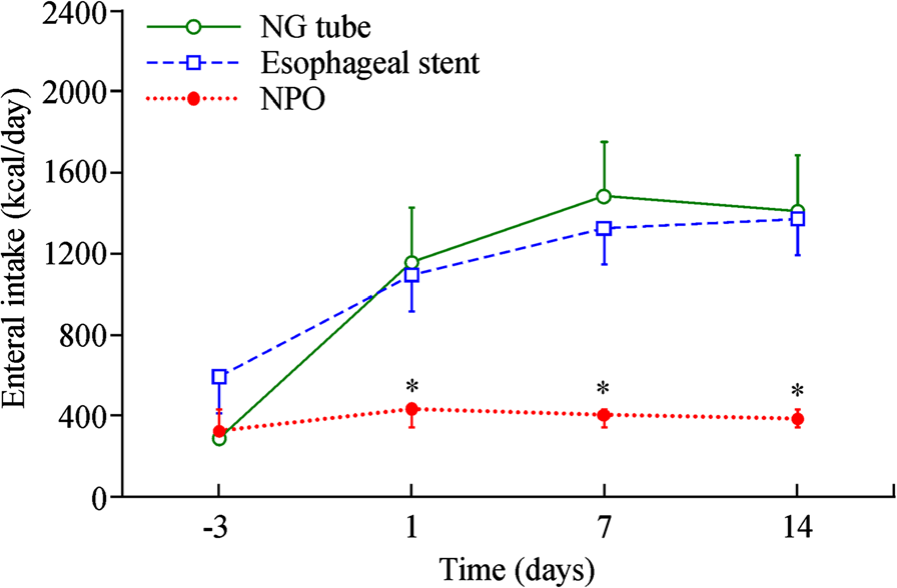
Daily enteral caloric intake for patients with an NG tube and those with an esophageal stent. Intake increased progressively in both groups. Each point and vertical bar represents the mean ± standard deviation. *P < 0.05

There was a significant difference in enteral caloric intake among the three groups (*p =* 0.01, Table 2), but no significant difference was found between the NG feeding tube group and the esophageal stent group. Serum albumin data were available for 11 of the 12 patients who received an NG feeding tube, 8 of the 10 patients who received an esophageal stent, and all 9 patients who were placed on an NPO regimen. Compared with baseline, serum albumin at 3–4 weeks was decreased only in the NPO group. There were significant differences in serum albumin among the three groups after the procedure (3.1 g/dL in the NG feeding group, 3.0 g/dL in the stent group, 2.1 g/dL in the NPO group; *p* < 0.01, Table 2). We recorded enteral caloric intake on the day before initiation of palliative treatment and on the first, third, seventh, and fourteenth days of treatment (Fig. 2). The calorie of enteral nutritional formula was one Kcal/ml.

### Survival

Median survival after diagnosis of malignant esophageal obstruction was 91 days overall: 122 days for the 12 patients who received an NG feeding tube, 133 days for the 10 patients who received an esophageal stent, and 51 days for the 9 patients who received supportive care under an NPO regimen. The length of hospital stay was 19 days in the NG group, 12 days in the esophageal stent group, and 39 days in the NPO group (Table 2). Compared to the NG feeding tube group and the esophageal stent group, the NPO group had significantly inferior overall survival (*p* < 0.01; Table 2). However, there was no significant difference in overall survival between the NG feeding tube group and the esophageal stent group (*p* = 0.56, Table 2). After adjustment for age, dysphagia, and presence of fistula, a Cox regression model found a significant difference among the three groups (Table 3). Patients in the NG group had an adjusted hazard ratio of 0.09 (95 % confidence interval 0.02–0.36), and patients in the stent group had an adjusted hazard ratio of 0.16 (95 % confidence interval 0.03–0.84). However, there was no significant difference in median overall survival between the NG feeding tube group and the esophageal stent group after adjustment for other prognostic factors.

**Table 3:** Crude and adjusted hazard ratios for overall survival

Group	Crude HR	95 % CI	Adjusted HR	95 % CI
NPO	Reference		Reference	
NG tube	0.16*	0.05–0.50	0.09*	0.02–0.36
Esophageal stent	0.12*	0.03–0.40	0.16*	0.03–0.84

Adjusted for age, fistula and dysphagia score

*p < 0.05. Comparisons are made using NPO group as reference

*NG tube* nasogastric tube, *NPO* nil per os, *HR* hazard ratio, *CI* confidence interval

**Fig. 3:**
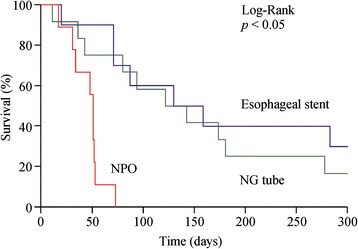
Survival curve plotted using Cox regression analysis

On multivariate Cox regression analysis, nutritional palliation by endoscopically guided NG tube placement *(p* = 0.03, hazard ratio 0.09, 95 % confidence interval 0.02–0.36) was an independent predictive factor of better overall survival in patients with advanced esophageal cancer (Table 3). Survival curves, adjusted for the Cox regression, were plotted for the tube and stent groups (Fig. 3)

### Complications

NG feeding tube placement was technically successful in all patients without severe procedure-related complications such as perforation, massive bleeding, or mortality. Similarly, stent placement was successful in all patients, without severe procedure-related complications. Two patients in the tube group and 3 in the stent group experienced post-procedural retrosternal pain and chest discomfort. The most common disease-related complication in the tube group was aspiration pneumonia (58 %), which also occurred in 5 patients (50 %) in the esophageal stent group and all 9 patients (100 %) in the NPO group; there was no significant difference in the incidence of aspiration pneumonia among the NG feeding tube group and the esophageal stent group (*p* = 0.63; Table 2). Following the procedure, 7 patients in the NG tube group (75 %) and 2 in the stent group (29 %) experienced dislodgment and required a repeat procedure. The average duration of dislodgment in NG tube group was 46.3 days. All repeated procedures for dislodgment were successful. Overall, there were no differences in the incidence of complications between the NG feeding tube group and the esophageal stent group, except for dislodgement (*p =* 0.01; Table 2).

## Discussion

The survival benefit of endoscopically assisted NG tube placement under fluoroscopy as compared with placement of a metal esophageal stent has not been well documented. Our study is the first report to compare median survival among three groups receiving respectively an NG tube, an esophageal stenting, and supportive care under an NPO regimen, as well as the first to reveal the significantly shorter median survival, in comparison with the stent and tube groups, of patients placed on an NPO regimen with only supportive care. Moreover, we are the first to observe the lack of significant difference in overall survival patients receiving an NG feeding tube and those receiving an esophageal stent.

Advanced esophageal cancer has a poor prognosis, with a 5-year survival rate of 10–15 %. Malignant esophageal obstruction resulting from such cancer can cause malnutrition, increases mortality risk, and reduces tolerance to radiotherapy and chemotherapy [13]. The harmful effect of cachexia, resulting from the obstruction, on severity of illness and mortality risk is of particular concern. An Asian retrospective study reported that the median overall survival of patients with AJCC stage IV cancer was 125 days [14]. Because advanced esophageal cancer is not curable, palliative treatment is required, particularly to improve quality of life and correct dysphagia and poor nutrition [15]. In fact, more than 50 % of patients with esophageal cancer have an inoperable condition at presentation and require palliation for poor nutrition [16]. Palliative care should result in a low complication rate in these patients and is usually performed during a limited hospital stay because their life expectancy is short [17].

European nutrition societies recommend using enteral nutrition as first-line support in patients with a functional gut. [18]. If this route is not available, then parenteral nutrition may be selected. However, parenteral nutrition should not be given regularly and a thorough assessment needs to be done before the initiation of parenteral nutrition. Full consideration of assessment includes patients’ clinical condition, psychosocial aspect and potential economic constraints [19]. When feeding routes are in place, it is easier to provide patients with medication in addition to nutrition and hydration. Feeding has a well-being purpose in some patients, particularly in the last period of life. Preserving nutrition and daily functional activity is one goal of enteral feeding, and relieving hunger and thirst is another goal. Refractory cachexia is characteristic of low performance score and expected survival of less than 3 months. If the patients are in refractory cachexia stage, therapeutic interventions, such as NG tube feeding or esophageal stenting, may alleviate the hunger and eating-related distress of patients and families [6]. Some evidence also supports clinical significance for the establishment of enteral nutrition for patients with esophageal cancer. Compared with total parenteral nutrition, enteral nutrition has been found to have a lower frequency of metabolic problems [20]. It is important for maintaining the normal mucosal barrier of the gut, which blocks the invasion of gut flora and prevents immune compromise caused by toxic side effects [21]. Endoscopically assisted NG tube placement under fluoroscopic guidance is a widely used way to establish enteral nutrition. However, instead of enteral nutrition, metal esophageal stenting, which has been used since the 1990s, is currently the main option to palliate malignant esophageal obstruction [22].

Homs et al. suggested that stent placement be reserved for patients with severe dysphagia and a short life expectancy or for those with poor response to brachytherapy; they found the median survival after stent placement to be 145 days [16]. Grilo et al. showed that percutaneous endoscopic gastrostomy (PEG) may be a suitable enteral nutrition technique in patients unsuitable for esophageal stenting, and they reported mean survival after PEG to be 5.9 months [23]. Ferraz et al. reported that overall median survival after gastrostomy for patients with advanced esophageal cancer was 61 days (range 1 to 551 days) [24]. When stenting, radiation, and PEG are technically not feasible, adequate caloric intake should be maintained by nutritional support with an NG feeding tube [25].

Advanced TNM stage, weight loss, dysphagia, fistula, and advanced age are known independent indicators of poor prognosis for patients with esophageal cancer [26]. In the present study, median overall survival of patients in the NPO group was approximately half that of patients in the NG group and esophageal stent group. A Cox regression model found significant differences in survival among the three groups with regard to age, dysphagia, and presence of fistula. However, no significant difference in median overall survival between the tube group and the stent group was demonstrated after adjustment for other potential prognostic factors.

Compared to the NG feeding tube group and the esophageal stent group, the NPO group had significantly longer length of hospital stay (*p =* 0.01; Table 2). After establishment of enteral feeding, the need of intravenous fluids became less. The administration of home parenteral nutrition in incurable cancer patients was a debate among different specialists and in the field of bioethics [27]. The patients with NG feeding or esophageal stent were cared more easily by their care-giver at home without intravenous fluids support.

Establishment of enteral nutrition is another predictor of median overall survival in patients with advanced esophageal cancer. It has many advantages as a treatment in patients with advanced malignant obstruction, including physiological response, immunocompetence, increased quality of life, and cost-effectiveness [28]. However, in our study, enteral nutrition and esophageal stenting produced similar benefits, with both improving overall survival. Lazaraki et al. reported that metal esophageal stenting was a superior choice for malignant esophageal dysphagia, with a low rate of procedure-related complications (0–17 %) and low procedure-related mortality (0–2 %) [29]. In the present study, the rate of procedure-related complications in both groups was very low, showing that endoscopy-assisted NG tube placement and metal esophageal stenting are safe even in patients who have been heavily treated or are in a late stage of disease. However, Nicolas et al. found the incidence of aspiration pneumonia in patients with an NG feeding tube to be 53 %, a result of gastroesophageal reflux related to the presence of the tube; in our study, the incidence of aspiration pneumonia in the tube group was 58 %, which is comparable. There was no significant difference in the incidence of aspiration pneumonia between the tube group and the stent group. However, the incidence of dislodgment in the tube group was significantly higher than in the stent group (58 % vs. 20 %, *p* = 0.01; Table 2). Food bolus obstruction and removal of the NG tube by the patient were the main reasons for this.

A 2004 study by Homs et al. showed that there was no significant difference in median survival between patients who underwent brachytherapy (155 days) and patients who underwent stent placement (145 days), although patients who underwent brachytherapy had better quality of life after a follow-up of at least 3 months [30]. However, brachytherapy may not be suitable for advanced esophageal cancer patients with a short life expectancy who need quick establishment of enteral nutrition or for patients who have previously failed brachytherapy [31]. NG tube placement and PEG should be reserved for patients with short life expectancy and those in poor medical condition. Although PEG is thought to be the better option for a long-term feeding route, NG tube placement and esophageal stenting may play a role when the endoscope cannot pass the obstruction site or when PEG fails. Shukla et al. reported a success rate of approximately 75 % for endoscopy-assisted NG tube placement [32]. In our study, NG tube placement was attempted in 14 patients. Two patients failed because of nearly complete obstruction of the tumor site and were treated with supportive care under an NPO regimen. As we used fluoroscopic guidance, our tube placement success rate was 86 %, an increase from that reported by Shukla et al. [32].

Squamous cell carcinoma of the esophagus is associated with lower socioeconomic status [33], and NG tube placement (US$235) costs less than esophageal stenting (US$2,205). Therefore, endoscopically assisted NG tube placement under fluoroscopic guidance might be an affordable alternative for patients who cannot afford a metal esophageal stent.

There are some limitations to our study. First, its retrospective design and small sample size mean that large prospective cohort studies are needed to confirm the impact of endoscopically assisted NG tube placement in patients with advanced malignant esophageal obstruction. Second, data on the patients’ symptoms and follow-up were obtained from reviews of medical records, which might introduce selection bias. Third, because the Taiwanese national health insurance system does not reimburse patients for metal esophageal stents, patients in the esophageal stent group might have had better socioeconomic status than those in the NG and NPO groups. Fourth, we did not assess quality of life. Metal esophageal stenting might be preferable for patients with a short life expectancy who require quick relief from dysphagia [30], as eating by mouth allows patients to taste the food, and this might be related to quality of life. Fifth, it was difficult to calculate total caloric intake from enteral source and parenteral source during hospitalization and home-care period. There was no significant difference (*p* = 0.08; Table 2) in the amount of intravenous fluids among the three groups. The patients who received peripheral parenteral nutrition (PPN) after establishment of the enteral route are two in the NG tube group, one in the esophageal stent group, and two in the NPO group. No patient received total parenteral nutrition. In the NG tube group and the esophageal stent group, parenteral nutrition was stopped one day after establishment of enteral route. One patient in NPO group received 1800 ml of PPN for 5 days and stopped PPN because of pulmonary edema. The other patient in NPO group received 1200 ml PPN for 16 days and stopped PPN because of patient and family’s decision. The average mean survival in NPO group with PPN was 62 days, which was superior to mean survival in NPO group. Insufficient nutrition support may be one of the reasons of poor prognosis in NPO group. The amount of enteral intake, however, was increased significantly in the NG tube group and the esophageal stent group. The procedure may also alleviate the hunger and eating-related distress of incurable cancer patients and families [6].

## Conclusion

In conclusion, both endoscopically assisted NG tube placement under fluoroscopic guidance and esophageal stenting significantly improved the survival of patients with malignant esophageal obstruction, with no significant difference between the treatments. Palliative enteral feeding by NG tube is safe, inexpensive, and has a low complication rate. Therefore, we suggest that endoscopy-assisted NG tube placement under fluoroscopic guidance can be considered as an alternative method of palliative treatment for malignant esophageal obstruction for patients who have a short life expectancy or who are unable to receive esophageal stenting due to cost concerns or personal choice.

## Additional file

Additional file 1:
**Research ethics approval.** (PDF 817 kb)

## Abbreviations

AJCC: American joint committee on cancer
NG: Nasogastric
NPO: Nil per os
PEG: Percutaneous endoscopic gastrostomy
