# Supplementary material for: Palliative enteral feeding for patients with malignant esophageal obstruction: a retrospective study
Source: BMC Palliat Care. 2015 Nov 5;14:58. doi: 10.1186/s12904-015-0056-5 (PMC4635529; doi:10.1186/s12904-015-0056-5)
Supplement: Additional file 1: — Research ethics approval. (PDF 817 kb) [file 12904_2015_56_MOESM1_ESM.pdf]

TEL: 886-2-87923311 ext 10552
FAX:886-2-2793-6049 / 2792-9201
E-mail: tsghirb@ndmctsgh.edu.tw

國防醫學院三軍總醫院
人體試驗審議會

11490 台北市內湖區成功路二段 325 號 醫療大樓五樓 5113 室
No. 325, Sec.2, Cheng-Kung Rd. Neihu 11490, Taipei, Taiwan, R.O.C

人體試驗計畫同意函

本審議會核准編號：2-104-05-030
計畫名稱：緩和性腸道營養對於惡性食道阻塞之研究
執行機構：三軍總醫院
計畫主持人：胃腸肝膽科張維國醫師
計畫書版本日期：V1.0_20141219
本會審核通過之其他文件版本及日期：中文摘要：1040428 個案報告書：V4_0_1031128
業經本院2015年04月30日人體試驗審議會第二審議會審查通過，並同意免受試者同意書執行，該計畫案經評估屬低度風險，(持續審查頻率為每年一次)，有效期限至2016年04月29日，特此證明。

本審議會的運作，遵循藥品優良臨床試驗準則及政府相關法律規章。計畫主持人應於同意函有效期屆滿前一個月，提出展延申請，本案須經本院人體試驗審議會通過後，方可繼續執行。

Letter of Approval
Institutional Review Board, Tri-Service General Hospital

TSGHIRB No.: 2-104-05-030
Protocol title: Palliative Enteral Feeding for Patients with Malignant Esophageal Obstruction.
Research institution: Tri-Service General Hospital
Principle investigator: Chang Wei-Kuo
Protocol version: V1.0_20141219
Other documents: Chinese Abstract：1040428；Case Report Form：V4_0_1031128
On 04/30/2015, the Institutional Review Board Ⅱ of the Tri-Service General Hospital approved the above-named application.
The board is organized and operated in compliance with International Conference on Harmonization (ICH) / WHO Good Clinical Practice (GCP) and applicable laws and regulations.
This approval is valid for 1 year till 04/29/2016. The principle investigator is required to submit the application for extension 1 month before the expiration date.

Institutional Review Board

Chairman 余慕賢 Yu Mu Hsien

三軍總醫院 TSGHIRB 人體試驗審議會
